# Coherent spontaneous resting EEG of frontal regions in human brain

**DOI:** 10.1186/1471-2202-13-S1-P25

**Published:** 2012-07-16

**Authors:** Shao-Wei Xue, Yu-Qin Deng, Yi-Yuan Tang

**Affiliations:** 1 Institute of Neuroinformatics, Dalian University of Technology, Dalian 116024, China; 2 Texas Tech Neuroimaging Institute and Dept of Psychology, Texas Tech University, Lubbock, TX79409, USA

## 

Recent research has demonstrated that spontaneous brain activity is not random. At the level of large-scale brain systems, the ongoing activity measured with functional MRI reflects the organization of many highly coherent functional networks [1]. Synchronization likelihood is a general measure of the temporal correlation between two time series sensitive to linear as well as non-linear statistical interdependencies [2]. However, how to use synchronization likelihood to detect the EEG activity of brain regions is still not clear. In the present work, EEG data was obtained from 17 healthy subjects (10 males, mean age 22.9) at rest with the eyes closed. Functional connectivity between brain activities at different electrodes was estimated using the synchronization likelihood method at four bands respectively [3]: theta (4 - 8 Hz)、 alpha1 (8 - 10 Hz)、 alpha2 (10 - 13 Hz) and beta (13 - 30Hz). Following the previous study [4], individual electrodes were grouped into the six regions: frontal (FP1, FP2, F7, F3, Fz, F4, F8, AF7, AF3, AF4, AF8, F5, F1, F2, F6), central (FC5, FC1, FC2, FC6, C3, Cz, C4, FC3, FC4, C5, C1, C2, C6), parietal (CP1, CP2, P3, Pz, P4, CP3, CPz, CP4, P1, P2), left temporal (T7, CP5, P7, FT7, TP7, P5), right temporal (T8, CP6, P8, FT8, TP8, P6), and occipital areas (O1, Oz, O2, PO7, PO3, POz, PO4, PO8). We found that the frontal group had larger mean synchronization likelihood than any other group at each band, showing high coherent spontaneous EEG of human brain. Mean synchronization likelihood between groups had no these trends.

## Conclusions

Synchronization likelihood could prove helpful for detection and estimation of functional relations between electrodes within and without brain regions.
